# Genetic Diversity Affects the Daily Transcriptional Oscillations of Marine Microbial Populations

**DOI:** 10.1371/journal.pone.0146706

**Published:** 2016-01-11

**Authors:** Irina N. Shilova, Julie C. Robidart, Edward F. DeLong, Jonathan P. Zehr

**Affiliations:** 1 Department of Ocean Sciences, University of California Santa Cruz, Santa Cruz, California, United States of America; 2 School of Ocean and Earth Science and Technology, University of Hawai’i at Manoa, Honolulu, Hawaii, United States of America; Laval University, CANADA

## Abstract

Marine microbial communities are genetically diverse but have robust synchronized daily transcriptional patterns at the genus level that are similar across a wide variety of oceanic regions. We developed a microarray-inspired gene-centric approach to resolve transcription of closely-related but distinct strains/ecotypes in high-throughput sequence data. Applying this approach to the existing metatranscriptomics datasets collected from two different oceanic regions, we found unique and variable patterns of transcription by individual taxa within the abundant picocyanobacteria *Prochlorococcus* and *Synechococcus*, the alpha Proteobacterium *Pelagibacter* and the eukaryotic picophytoplankton *Ostreococcus*. The results demonstrate that marine microbial taxa respond differentially to variability in space and time in the ocean. These intra-genus individual transcriptional patterns underlie whole microbial community responses, and the approach developed here facilitates deeper insights into microbial population dynamics.

## Introduction

Marine microbial communities play critical roles in the cycling of organic matter and nutrients and in food webs [1,2]. These complex communities are composed of diverse, poorly-characterized taxa, including many uncultivated lineages and strains [3–6]. Much has been learned about marine microbial communities using cultivation-independent methods, in particular, metagenomics and metatranscriptomics approaches enabled by high-throughput and next-generation nucleic acid sequencing [5,6–10]. One recent discovery is that individual populations of phototrophic and heterotrophic plankton have complex but coordinated rhythmic daily transcription patterns that are highly conserved throughout oceanic microbial communities worldwide [11–13]. This was particularly surprising, since genome comparisons of closely-related cultivated and uncultivated taxa, such as the abundant phototrophs *Prochlorococcus* and *Synechococcus*, show that strains or ecotypes are diverse and variable in nucleotide sequence and gene content [10,14–18]. Studies with cultured isolates showed that genotypic diversity among bacterial strains is reflected in gene regulation at the transcription and expression levels [19,20], and gene regulation has been recognized as an important factor driving evolution and adaption in microbes [21]. It is currently not clear how the potentially great physiological diversity implied by high genomic variability in marine microbial populations [5] could result in consistent, robust and synchronized daily gene transcription patterns in the environment.

The whole genome population binning (WGPB) approaches [11–13] for analysis of the high-throughput sequence data have the advantage of examining transcriptional responses across the whole genome, but without appropriate reference genomes, cannot usually resolve populations at the species or sub-species taxonomic levels. This high resolution is only possible when genome sequences are known and metagenomic/metatranscriptomic datasets have high coverage for these genomes [9]. At the current depth of sequencing, assigning short reads to individual taxa is only possible for taxonomically identifiable signature regions and for genes/transcripts present in high abundance (for example, the <200 nucleotide long region of the proteorhodopsin gene has been examined in detail in order to evaluate *Pelagibacter* subtaxa contributions in [11]). Oligotyping [22] and minimum entropy decomposition [23] are powerful approaches that uncover finer community changes based on a marker gene, but both require that sequences cover the same region of the gene (for example, the V4-V5 hypervariable region of the 16S-rRNA gene). We developed a microarray-inspired gene centric (MAGC) approach that links sequences originated from different regions of a gene/transcript and resolves transcription of closely-related taxa. We applied MAGC to examine the contribution of individual microbial taxa to synchronized transcription patterns observed previously at the genus level [11,12] and examined transcription of specific functional genes by individual taxa within the cyanobacteria *Prochlorococcus* and *Synechococcus*, the proteorhodopsin-containing alpha proteobacterium *Pelagibacter ubique* (SAR11) and the eukaryotic picophytoplankton *Ostreococcus*. The results showed that closely related microbial taxa (defined as taxa that have greater than 95% nucleotide identity to the target gene sequence) have distinct and differential transcription patterns for ecologically-relevant functional genes, and these individual patterns may reflect the differences in microbial responses to environmental conditions.

## Results and Discussion

**Fig 1:**
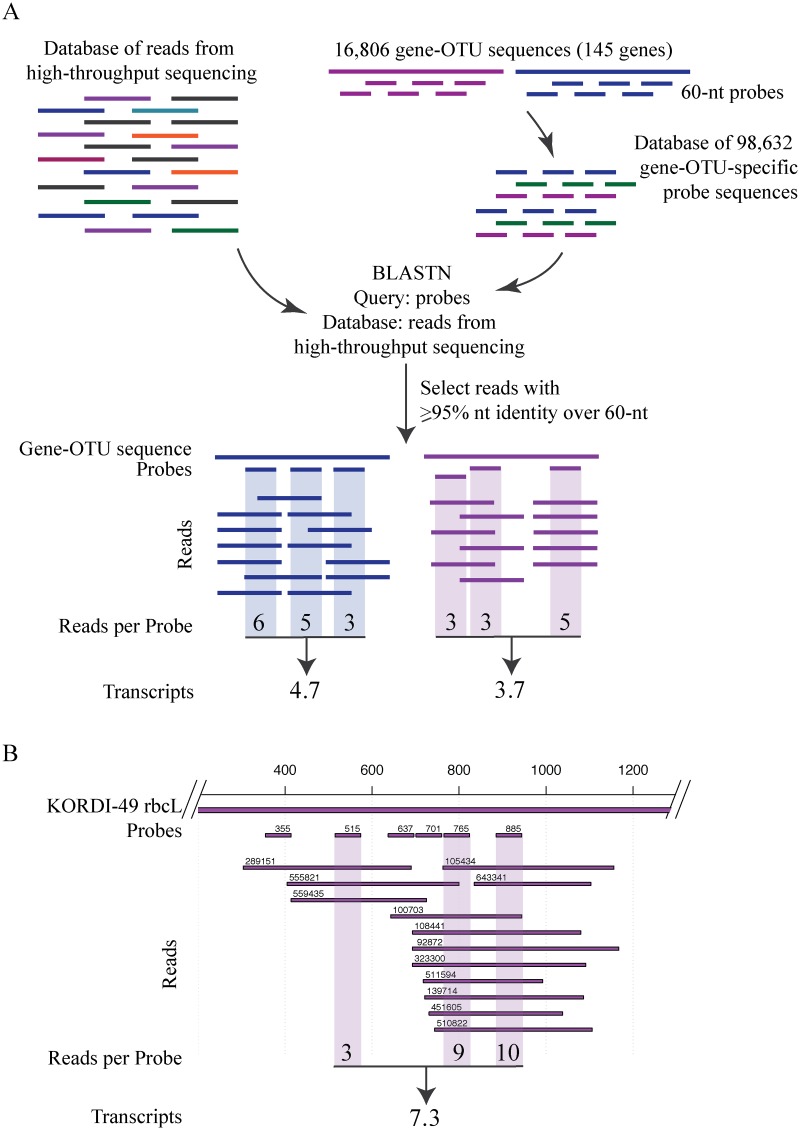
Principle of the MAGC approach for analysis of nucleotide sequences obtained from high-throughput sequencing. (A) Each of the16,806 gene-OTU has six highly specific probes, and all of the 98,632 probe sequences are used as queries in BLASTN searches against the database of nucleotide sequences obtained from environmental samples (metagenomes or metatranscriptomes). The transcript-OTU abundances are estimated as the averages of reads with ≥95% nucleotide identity (nt) across the entire length of each probe. (B) Distribution of probes specific for the *Synechococcus* KORDI-49 *rbcL* gene shown with reads identified with these probes in a California Current System diel study. Three probes (indicated with shaded area) for *Synechococcus* KORDI-49 *rbcL* had ≥95% nt sequence identity to reads from this sample. The read ID and probe start position are shown above each read and probe, respectively.

The MAGC approach described in this study (Fig 1) is an in silico nucleotide sequence-query based method that uses 60-nucleotide (nt) long sequences (probes) previously designed for the MicroTOOLs environmental microarray [24]. The probes are specific for known marker genes for metabolic and cellular processes important for microbes living in pelagic oligotrophic environments. These processes include energy, carbon and nitrogen metabolisms, phosphonate and dimethylsulfoniopropionate utilization, nutrient transport, stress responses, DNA replication and cell division [25–30]. The ortholog sequences for each marker gene were collected from marine metagenomic and metatranscriptomic sequence datasets and marine microbial genome sequences. These sequences were clustered at 95% nucleotide identity, and a total of six probes were designed for each representative target sequence [24]. Thus, the ~99,000 probes that are specific for 16,800 orthologs of 145 different functional genes target a diverse set of representative organisms and genes from pelagic microbial communities. In the MAGC approach, this MicroTOOLs probe set was used as a query set against metatranscriptomic sequence data to discriminate gene transcription at a high phylogenetic resolution (Fig 1). In this paper, we define a group of gene sequences or transcripts that shares 95% nt identity as a gene-Operational Taxonomic Unit (OTU) or transcript-OTU, respectively. Because six probes target the same individual transcript-OTU, and transcript abundance is estimated as the average of all probe hits (Fig 1), the MAGC approach links several reads over the length of the transcript to one transcript-OTU and shows OTU-specific patterns in gene transcription.

We used the MAGC approach to analyze previously published metatranscriptomic datasets that were obtained from a three-day duration, two-hour interval sampling in the North Pacific Subtropical Gyre (NPSG) [12] and a two-day duration, four-hour interval sampling in the California Current System (CCS) [11]. Both studies collected microbial biomass in the 0.2–5.0 μm size fraction from 23 m depth using the free-drifting robotic Environmental Sample Processor [11,12]. The CCS study consisted of 13 samples that were sequenced with a 454 GS FLX Titanium DNA sequencer (19 runs of 4.4 Gbp total, 450 bp long single reads), and the NPSG study consisted of 30 samples sequenced with an Illumina MiSeq sequencer (119 runs of 18.8 Gbp total, 150 bp long paired-end reads). The goal of this study was to investigate the transcriptional patterns of individual gene-OTUs within the cyanobacteria *Prochlorococcus* and *Synechococcus*, the alpha proteobacterium *Pelagibacter* and the eukaryote *Ostreococcus* during the diel cycle. The probes identified 0.68M (0.55% of total) and 0.38M (0.38% of total) sequence reads at 95% sequence identity in metatranscriptome data from the NPSG and CCS, respectively (Table A in S1 File). The number of identified reads per gene identified with MAGC were highly correlated (Pearson’s r 0.92±0.07, n = 3) with read counts identified by the WGPB approach in [11,12]. A large fraction of the reads per gene were identified by MAGC, with the median of 53% of reads per gene per sample identified by MAGC to reads identified by WGPB (Tables 1 and 2). Interestingly, the probes used in MAGC were originally designed for sequences that were available publically before 2010 and obtained from samples collected from different regions of the oceans before 2008 [24]. A large portion of the sequences came from the metagenomic dataset from Station ALOHA in the NPSG collected in 2002 [7], the Global Ocean Sampling expedition collected during 2004–2006 [8,31], and metatranscriptomic datasets from the North Pacific, Southwest Pacific and Equatorial Atlantic Oceans collected during 2005–2007 [32–34]. The CCS and NPSG datasets were obtained in 2009 (CCS) and 2011, respectively [11,12]. The fact that the probes captured a significant fraction of the targeted transcripts from the CCS and NPSG datasets and the high correlation between the MAGC and WGPB methods imply that, perhaps, we have now largely characterized the diversity of sequences in the environment, at least at the current depth of sequencing, making it feasible to use more targeted approaches, such as MAGC, to address hypothesis-driven ecological questions.

**Table 1:** Genes With Differential Transcription Patterns Among *Prochlorococcus* and *Pelagibacter* OTUs in the NPSG diel study.

Gene	Annotation	KEGG Pathway	Number of reads detected by MAGC		Pearson's r	OTU-transcripts detected
			Total reads	% of WGPB [12]		
***Prochlorococcus* in NPSG**						
*phoH*	phosphate starvation-inducible protein	[Phosphorus stress response]	2322	129	0.82	11
*sbtA*	sodium-dependent bicarbonate transporter	[Transporters (carbon fixation)]	481	46	0.91	4
*metC*	cystathionine beta-lyase family protein (aluminum resistance)	Amino acid metabolism	340	84	0.65	3
*dxs*	1-deoxy-D-xylulose-5-phosphate synthase	Carbohydrate and lipid metabolism	1522	48	0.96	15
*ppc*	phosphoenolpyruvate carboxylase	Carbon metabolism (carbon fixation)	1264	37	0.94	14
*icd*	isocitrate dehydrogenase	Carbon metabolism (citrate cycle)	601	51	0.93	8
*pyk*	pyruvate kinase	Carbon metabolism (glycolysis)	783	46	0.92	2
*prsA*	ribose-phosphate pyrophosphokinase	Carbon metabolism (pentose phosphate pathway)	1950	43	0.95	19
*zwf*	glucose-6-phosphate dehydrogenase	Carbon metabolism (pentose phosphate pathway)	590	56	0.97	12
*ftsZ*	cell division protein FtsZ	Cellular processes	502	12	0.94	5
*ndhI*	NADH dehydrogenase subunit I	Energy metabolism (oxidative phosphorylation)	1589	121	0.97	8
*petF*	ferredoxin	Energy metabolism (photosynthesis)	2099	91	0.91	6
*psaA*	photosystem I P700 chlorophyll a apoprotein A1	Energy metabolism (photosynthesis)	19081	76	0.95	43
*psbB*	photosystem II PsbB protein (CP47)	Energy metabolism (photosynthesis)	11960	66	0.97	47
*dnaA*	chromosomal replication initiator protein DnaA	Replication and repair	390	38	0.95	10
*dnaE*	DNA polymerase III subunit alpha	Replication and repair	139	7	0.76	3
*recA*	recombinase A	Replication and repair	5004	68	0.99	13
*rpoD*	RNA polymerase primary sigma factor	Transcriptional machinery	1027	29	0.99	21
*sigAII*	type II alternative sigma-70 family RNA polymerase sigma factor	Transcriptional machinery	1181	50	0.97	29
*pstS*	phosphate-binding transporter protein PstS	Transporters [Phosphorus stress response]	1479	32	0.97	3
*gidA*	glucose inhibited division protein A	tRNA modification factors	501	45	0.93	4
**AVERAGE**				**56±31**	**0.92±0.08**	
**SUM**	**21 genes**					**280**
***Pelagibacter* in NPSG**						
*bop*	proteorhodopsin		2699	7.3	0.94	12

For each gene, number of reads identified using MAGC for all samples and as a percent relative to the number of reads identified by WGPB [12], correlation (the Pearson correlation coefficient) between results obtained by the two approaches and number of transcript-OTUs identified in all samples are shown. In the KEGG pathway column, the square brackets indicate pathways/metabolisms/processes that do not have KEGG assignment.

**Table 2:** Genes With Differential Transcription Patterns Among *Synechococcus*, *Pelagibacter* and *Ostreococcus* OTUs in the CCS diel study.

Gene	Annotation	KEGG Pathway	Number of reads detected by MAGC		Pearson's r	OTU-transcripts detected
			Total reads	% of reads by WGPB [11]		
***Synechococcus* in the CCS**						
*sodN*	putative nickel-containing superoxide dismutase	[Oxidative stress response]	47	55	0.88	2
*ppc*	phosphoenolpyruvate carboxylase	Carbon metabolism (carbon fixation)	30	48	0.79	3
*rbcL*	ribulose-bisphosphate carboxylase large chain	Carbon metabolism (carbon fixation)	617	82	0.97	7
*prsA*	ribose-phosphate pyrophosphokinase	Carbon metabolism (pentose phosphate pathway)	28	48	0.91	2
*coxA*	cytochrome c oxidase subunit I	Energy metabolism (oxidative phosphorylation)	91	56	0.96	5
*ndhI*	NADH dehydrogenase subunit I	Energy metabolism (oxidative phosphorylation)	73	135	0.89	5
*cpaB1*	phycocyanin, beta subunit	Energy metabolism (photosynthesis)	592	94	0.99	3
*cpc*	phycocyanin, beta subunit	Energy metabolism (photosynthesis)	54	9	0.82	2
*petF*	ferredoxin	Energy metabolism (photosynthesis)	74	128	0.96	6
*psaA*	photosystem I P700 chlorophyll a apoprotein A1	Energy metabolism (photosynthesis)	370	27	0.92	4
*psaB*	photosystem I P700 chlorophyll a apoprotein A10	Energy metabolism (photosynthesis)	352	51	0.95	7
*psbA*	photosystem II PsbA protein (D1)	Energy metabolism (photosynthesis)	189	47	0.97	6
*psbB*	photosystem II PsbB protein (CP47)	Energy metabolism (photosynthesis)	115	25	0.81	6
*recA*	recombinase A	Replication and repair	49	41	0.9	2
*rpoD*	RNA polymerase primary sigma factor	Transcriptional machinery	14	26	0.85	12
*sigAII*	type II alternative sigma-70 family RNA polymerase sigma factor	Transcriptional machinery	118	32	0.98	13
**AVERAGE**				**57±36**	**0.91±0.07**	
**SUM**	**16 genes**					**85**
***Pelagibacter* in CCS**						
*bop*	proteorhodopsin		950	32.1	0.90	10
*idiA*	iron deficiency induced protein A, ferric iron transporter		183	27.0	0.93	2
***Ostreococcus* in CCS**						
*rbcL*	RuBisCO, large subunit	Carbon metabolism (carbon fixation)	3649	29.0	0.96	2

For each gene, number of reads identified using MAGC for all samples and as a percent relative to the number of reads identified by WGPB [11], correlation (the Pearson correlation coefficient) between results obtained by the two approaches and number of transcript-OTUs identified in all samples are shown. In the KEGG pathway column, the square brackets indicate pathways/metabolisms/processes that do not have KEGG assignment.

**Fig 2:**
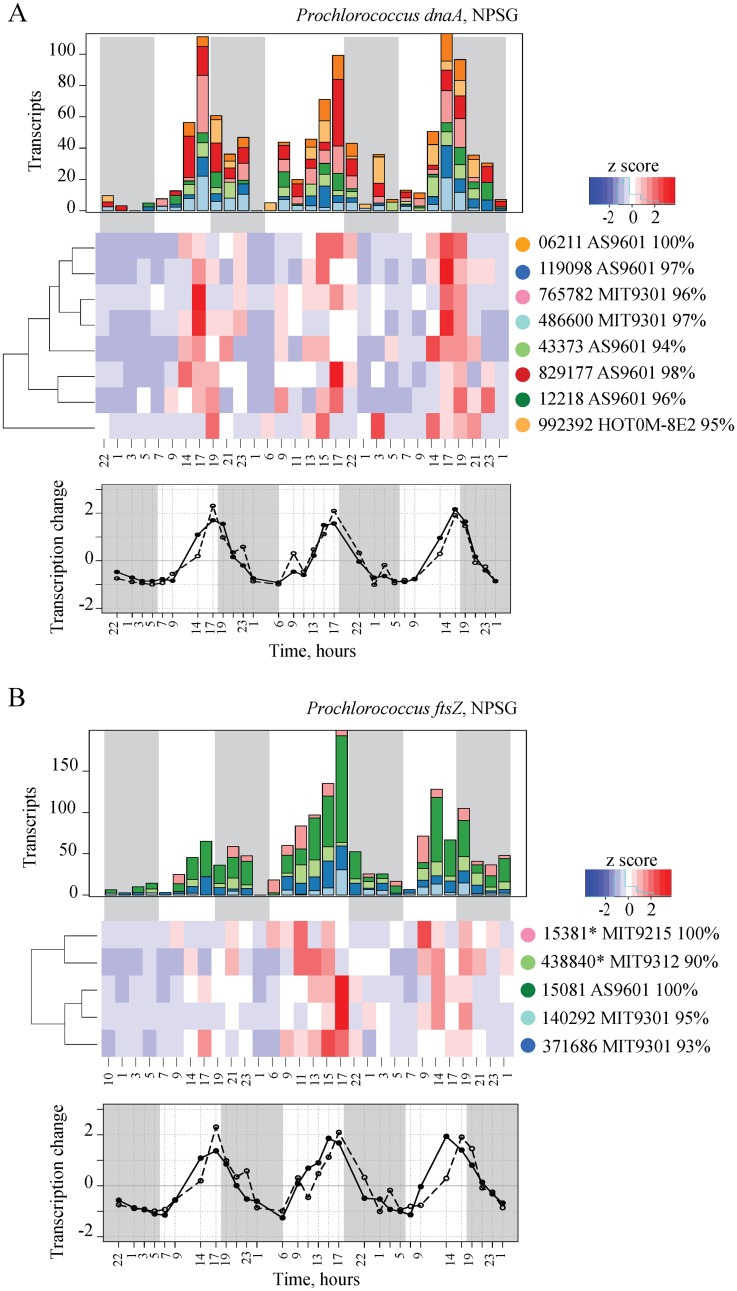
OTU-Specific *Prochloroccus* Daily Gene Transcription Patterns in the NPSG. Periodic transcription of the DNA replication initiation protein *dnaA* (A) and cell division protein *ftsZ* (B) genes varied among *Prochlorococcus* OTUs. Top panels: Transcriptional composition detected based on the MAGC approach, where transcription was normalized to the total *Prochlorococcus* hits in each sample, over time of day (X-axis in hours). OTUs are color-coded according to the heatmap. The transcript-OTU name (for example, 119098 AS9601 98%) shows ID of the target sequence (119098) for which probes were designed and percent nucleotide identity of the OTU to the most similar genome sequence (AS9601 98%). Transcription for all OTUs shown was estimated based on at least three probes with the exception of the two OTUs indicated with *. Middle panels: Hierarchical clustering of transcription patterns (by Pearson correlation). Each row in the heatmap shows transcription pattern of a unique OTU, and each column is a time point within the time-series. Bottom panels: Temporal patterns of total transcript abundances detected by MAGC (open circle) in this study and by WGPB (closed circle) [12] shows that the results of the two approaches are consistent.

The MAGC approach revealed that oscillating patterns of gene transcription in *Prochlorococcus* in the NPSG [12] were composed of distinct OTU-specific patterns (Fig 2). Seven hundred ninety-one unique *Prochlorococcus* transcript-OTUs were detected which represented 54 different genes (Table A in S1 File), and 55.6% of *Prochlorococcus* transcript-OTUs representing 42 genes had significant diel periodicity in their abundance. From the 42 periodically expressed genes, orthologs of 21 genes (63.5% of the periodic transcript-OTUs) had differential transcriptional patterns among *Prochlorococcus* taxa and included genes for DNA replication and repair and carbohydrate metabolism (Table 1). For example, the time of maximum transcription of the genes for the DNA replication initiation protein *dnaA* and for the key cell division protein *ftsZ* was distinct even among closely related high-light *Prochlorococcus* OTUs (Fig 2). This diversity in gene expression patterns was not resolved within binned *Prochlorococcus* populations using the WGPB approach, where transcription of both *dnaA* and *ftsZ* had periodic patterns with peaks at ~17 h and 16 h, respectively [12]. In contrast to genes with different patterns of transcription among gene-OTUs (Table 1), 16 genes had the same transcriptional patterns for all detected *Prochlorococcus* gene-OTUs (Table B in S1 File and S1 Fig). *Prochlorococcus* populations are comprised of genetically different strains that vary substantially in gene content [10,35,36]. Variations in the transcriptional patterns of genes such as DNA replication and cell division may reflect the differences in physiology, growth and cell division among these *Prochlorococcus* strains. Additionally, the fact that many genes with periodic transcription patters had differential transcription at the OTU level suggests differences in global regulatory mechanisms among the *Prochlorococcus* OTUs.

**Fig 3:**
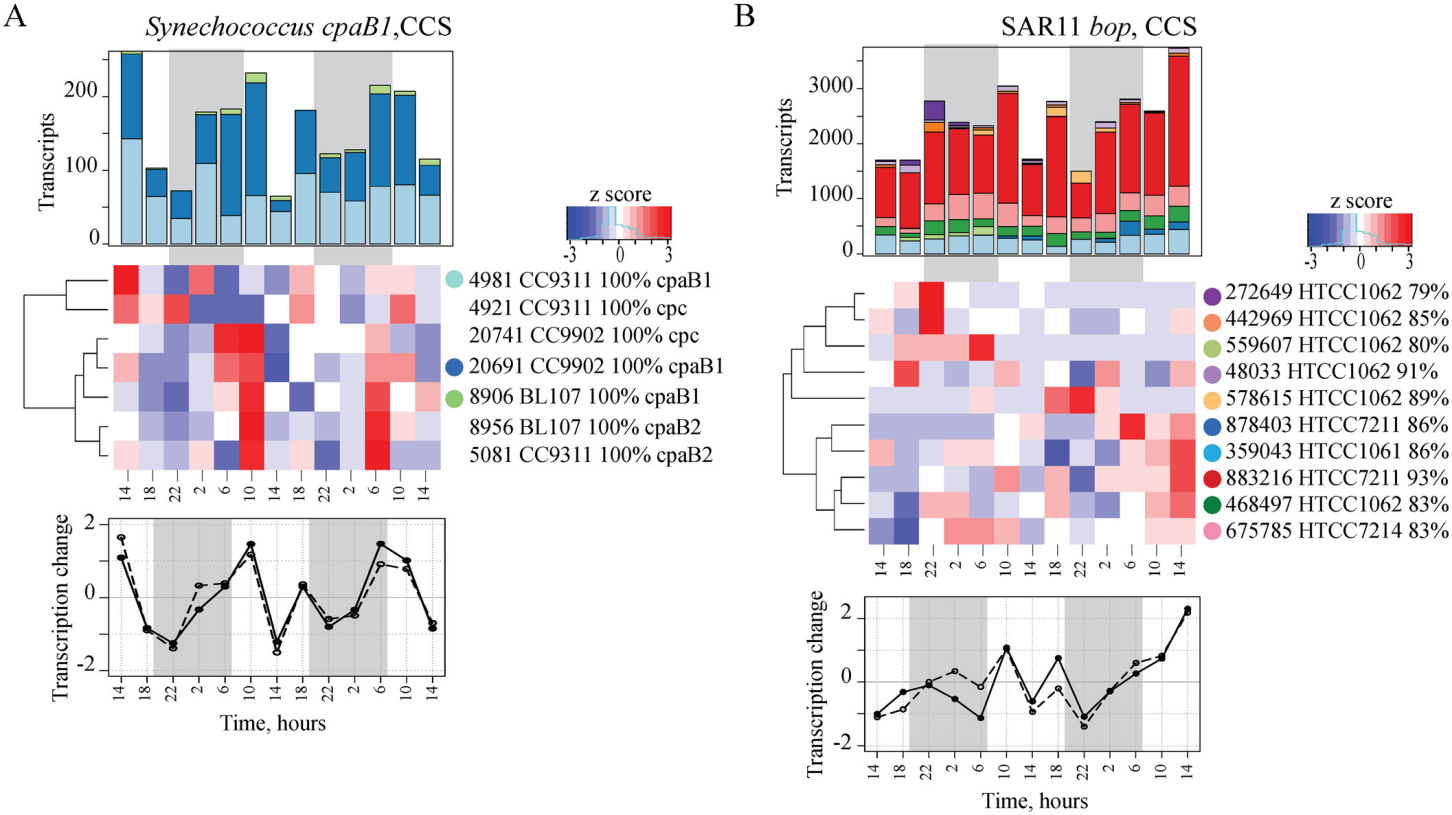
OTU-Specific Daily Gene Transcription Patterns by *Synechococcus* and SAR11 in the CCS. Periodic transcription of phycoerythrin type I *cpaB1* gene (A) varied among *Synechococcus* OTUs. Transcription of the *bop* gene varied among the SAR11 OTUs (B). Top panels: Transcriptional composition detected based on the MAGC approach, where transcription was normalized to the total *Synechococcus* (A) and SAR11 (B) hits in each sample, over time of day (X-axis in hours). OTUs are color-coded according to the heatmap. Middle panels: Hierarchical clustering of transcription patterns (by Pearson correlation). The heatmap for *Synechococcus* gene-OTUs (A) also shows transcriptional patterns for phycoerythrin type II *pcaB2* and phycocyanin *cpc* genes. Each row in the heatmap shows transcription pattern of a unique OTU, and each column is a time point within the time-series. Bottom panels: Temporal patterns of total transcript abundances detected by MAGC (open circle) in this study and by WGPB (closed circle) [11] shows that the results of the two approaches are consistent.

Daily transcription patterns also varied among *Synechococcus* gene-OTUs in coastal California Current waters. Among 98 unique transcript-OTUs for 37 different genes in *Synechococcus* (Table A in S1 File), 63.6% of the transcript-OTUs had differential patterns over the diel cycle (Table 2). Eighteen transcript-OTUs from ten different genes had significant periodic patterns, and two genes had significantly different periodic transcriptional patterns among the *Synechococcus* OTUs. Interestingly, transcription of the *cpc* gene encoding the pigment phycocyanin and the *cpaB1* gene encoding the type 1 phycoerythrin pigment had significant periodicity in the *Synechococcus* CC9902-like OTUs (e.g. *cpaB1* sequence is within 95% nucleotide identity to *cpaB1* sequence in cultured *Synechococcus* CC9902), but not in the co-existing *Synechococcus* CC9311-like OTU. The timing of maximum abundance of transcripts of the *cpc* and *cpaB1* genes was distinct for each OTU (Fig 3A). The *Synechococcus* OTUs also varied in time of peak transcript abundances for other genes including the key enzyme for carbon fixation, ribulose-bisphosphate carboxylase large subunit gene (RuBisCO) (*rbcL*; S2A Fig) and the photosystem I subunit gene *psaA* (S2B Fig). The *Synechococcus* CC9311, CC9902 and BL107-like strains detected in the CCS diel study ([11] and here) are members of two abundant and dynamically co-existing clades of *Synechococcus*, Clade I (CC9311) and Clade IV (CC9902 and BL107), and both clades chromatically adapt by changing their pigment content to optimize light utilization [37]. The different daily transcription patterns for genes encoding pigments, RuBisCO and photosystem I indicate that the two *Synechococcus* clades were responding differentially to temporal and spatial variability of environmental factors such as light and nutrient availability [37–39]. In addition, distinctions in *cpaB-1* and *cpc* transcription patterns among *Synechococcus* strains explains the lack of significant periodic patterns observed for these genes when clustered at the genus level [11].

The alpha proteobacteria SAR11 proteorhodopsin gene (*bop*) had a predominantly oscillating transcriptional pattern (with some variation) at the genus level [12], and the MAGC approach showed that these patterns varied substantially among different SAR11 OTUs in both the CCS and NPSG (Fig 3B, S3 Fig). In the NPSG, individual *bop* transcript-OTUs peaked at 3AM, 9AM and 11AM. Out of a total of eight *bop* transcript-OTUs, one had a weak periodic diel pattern (false discovery rate FDR = 0.23); however, patterns of most of the *bop* transcript-OTUs were dissimilar (S3 Fig), with Pearson correlation coefficients ranging from -0.19 to 0.60 (median of 0.35; n = 28). In the CCS, the *bop* pattern observed for the combined SAR11 populations was driven largely by the dominant transcript-OTU (40–60% of detected *bop* transcripts) (Fig 3B). The less abundant *bop* transcript-OTUs, comprising the remaining 40–60% of the transcripts, had oscillating peak transcript abundances. Thus, OTU-specific transcription demonstrates that one dominant OTU can mask detection of variability in individual patterns. The variable proteorhodopsin gene transcriptional patterns among OTUs suggest that genetic diversity among SAR11 strains [40] sometimes results in different daily gene transcription patterns. Proteorhodopsin is a light-driven proton motive pump, and while the effect of light on the transcription of the *bop* gene is debatable [41,42], proteorhodopsin is involved in SAR11 cell survival under carbon-limited conditions [43]. Thus, variation in *bop* transcript abundances among OTUs may reflect the differences in the metabolic status of the cells and reflect variability in environmental conditions (e.g. organic carbon concentration and chemical composition).

**Fig 4:**
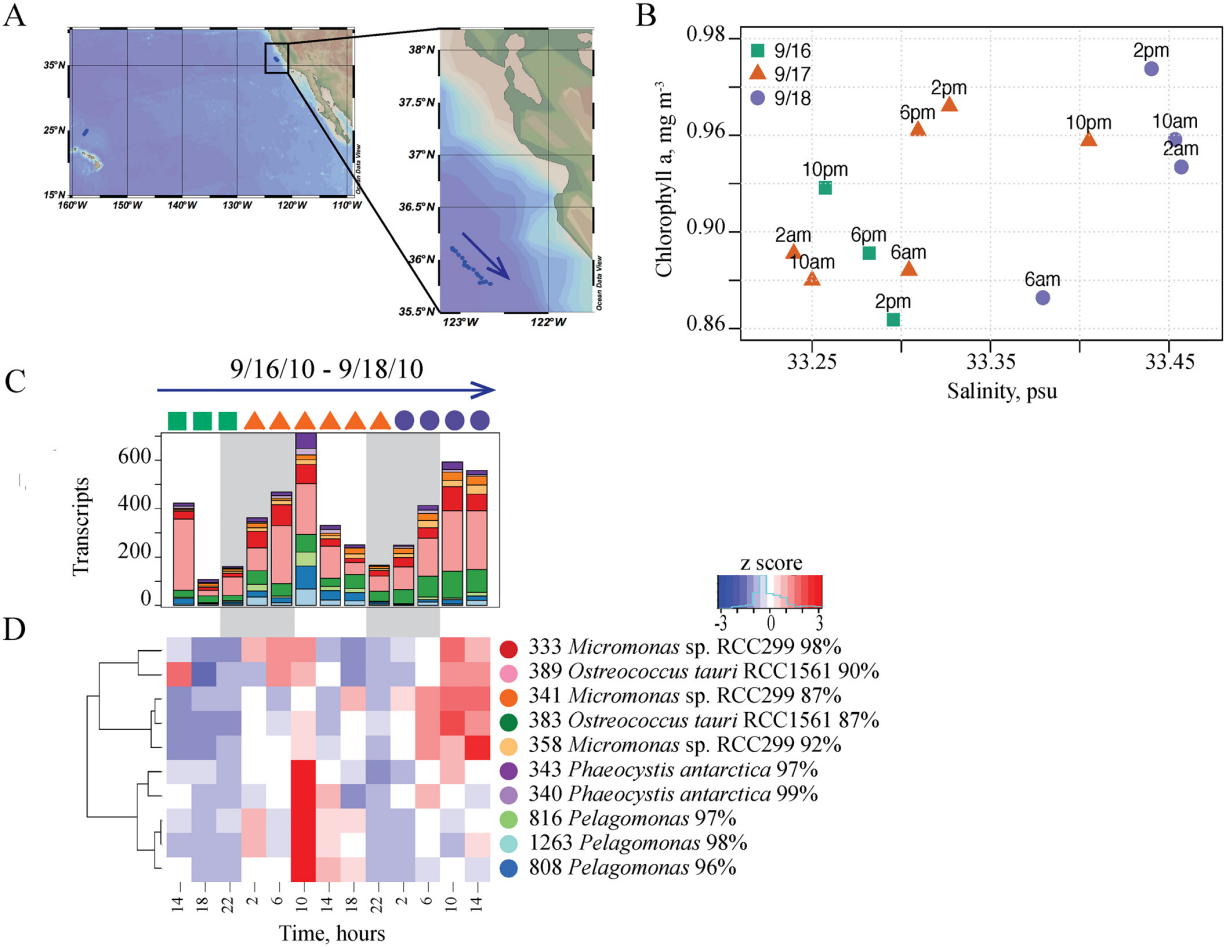
Eukaryotic Phytoplankton RuBisCO Gene (*rbcL*) Transcription Patterns in the CCS Associated with the Change in Water Masses. (A) Location and transect of the CCS diel study [11]. CTD cast stations that followed the drifting sampler are shown. (B) Chlorophyll *a* as a function of salinity during the period of the CCS transect showing that samples were taken from different water masses [11]. (C) Transcript composition and (D) transcriptional patterns of the *rbcL* gene by eukaryotic phytoplankton. Arrow indicates the direction of the drifting sampler.

Transcripts recovered in the CCS were dominated by the eukaryotic phytoplankter *Ostreococcus*, which displayed significant transcriptional periodicity across many genes, including the *rbcL* gene encoding the large subunit of RuBisCo [11]. OTU-specific transcriptional patterns showed that there was a change in the relative composition of the picoeukaryote *Ostreococcus* populations (Fig 4). We detected two different *Ostreococcus rbcL* OTUs (Fig 4C and 4D), with 87% and 90% similarity to the chloroplast of *O*. *tauri* RCC1561, and both transcript-OTUs had similar patterns of abundances during the CCS study (Pearson 0.74). However, while transcripts from one *Ostreococcus rbcL*-OTU were very abundant over the two days sampled, transcripts from another OTU-*rbcL* increased by more than a factor of two in relative abundance by the end of sampling period. Transcription of *rbcL* by other eukaryotic phytoplankton also shifted, with the relative abundances of haptophyte and stramenopile transcripts decreasing significantly on the second day (Fig 4D). These transcript shifts coincided with a change in water masses by the end of the diel sampling in the CCS [11] (also in Fig 4B). Thus, the shift in *rbcL* transcript abundances indicate OTU-specific responses to changed conditions (for example, nutrient availability) [44] and the advection of new populations with different water masses.

**Fig 5:**
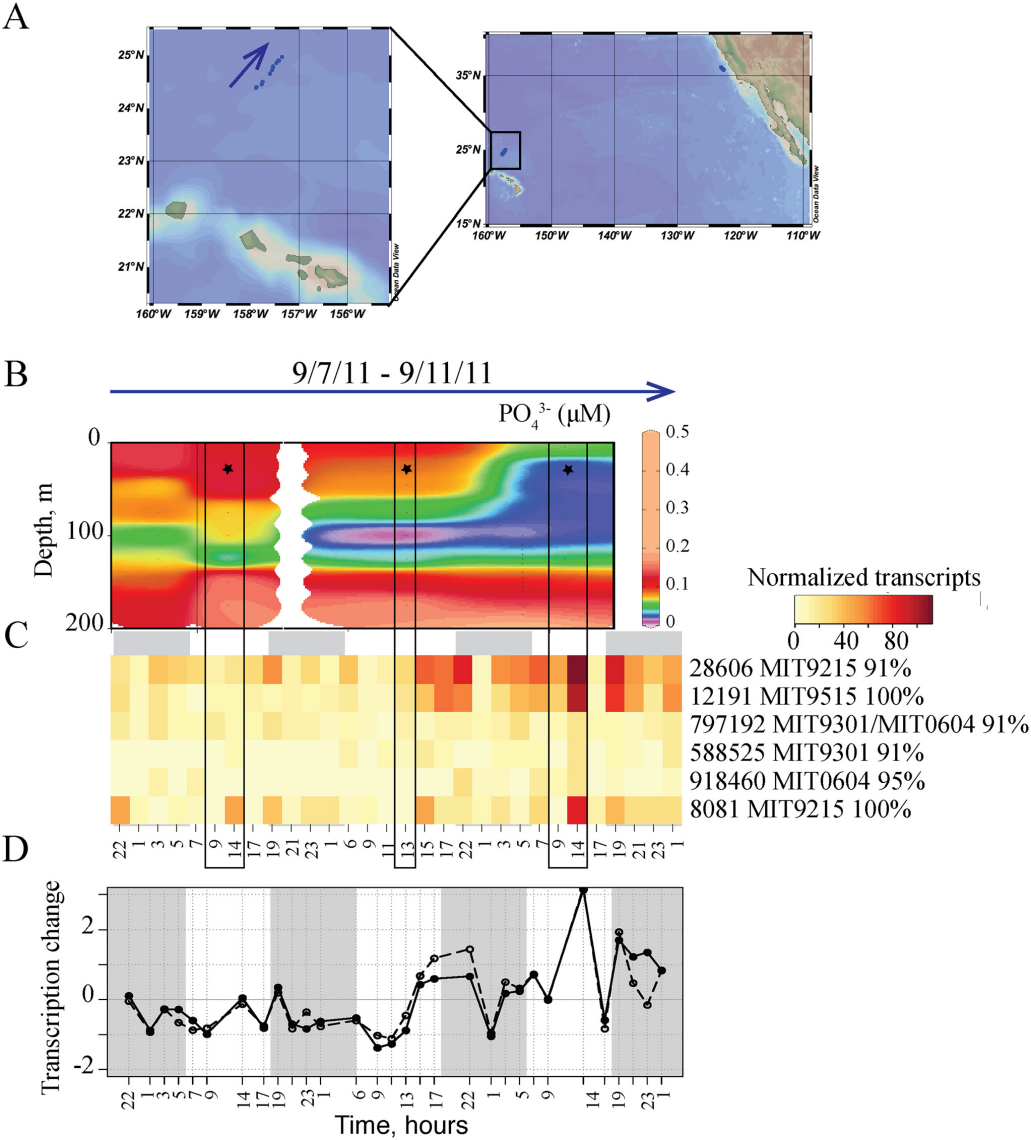
Transcription of the Phosphate Transporter Gene *pstS* by Specific *Prochlorococcus* OTUs Associated with Inorganic Phosphate Concentrations in the NPSG Study. (A) Location and transect of the NPSG diel study [12,45]. CTD cast stations following the drifting sampler are shown. (B) Phosphate concentrations measured from seawater collected from a CTD Niskin bottle following the drifting sampler are shown for the NPSG transect [45]. Eight depths were sampled at each of the five sampling locations, and the stars indicate 25 m depth. The boxes indicate the midday time of depth profile sampling for phosphate. (C) Normalized *pstS* transcript abundances among *Prochlorococcus* OTUs during the 72h NPSG study. (D) Temporal patterns of total *Prochlorococcus pstS* transcript abundances detected by MAGC (open circle) here and by WGPB (closed circle) [12].

Lagrangian sampling efforts, designed to sample the same water mass, are becoming more feasible to implement at sea using advanced robotic approaches, but deconvoluting the effects of spatial and temporal variability is still difficult. The metatranscriptomic samples [11,12] were collected with a robotic drifting sampler to approximate Lagrangian sampling, but the path of each drifter migrated between submesoscale features, with corresponding changes in salinity and other environmental conditions such as nutrient concentrations [11,45]. Differences in *pstS* (the gene encoding the high-affinity phosphate-binding protein that is a marker for phosphorus stress) transcript abundances along the NPSG sampling transect were particularly striking, with *Prochlorococcus pstS*-OTUs within 95% identity to sequences from MIT9215 and MIT9515 having highest transcript abundances in low-phosphate waters (Fig 5). Phosphorus is an important nutrient that can limit primary productivity in the ocean (Table D in S1 File, [45,46]), and these *pstS* OTU-specific patterns indicate that MIT9215 and MIT9515-like OTUs may be particularly sensitive to low phosphorus concentrations, relative to other high-light *Prochlorococcus* strains. Such OTU or strain-level differences are indicative of the specific niches occupied by different populations that lead to differences in growth rates as a function of phosphate concentration. Thus, the OTU-specific transcriptional patterns observed here reveal the differences in physiological statuses of co-existing members of the microbial community and their individual responses to environmental variability.

## Conclusions

The variability in transcription at the OTU or strain-level resolution helps explain how such genetically diverse, sympatric microbial communities [5,10] still retain robust daily patterns in gene transcription [13]. The OTU-specific patterns of gene transcription reported here show that closely-related but genetically distinct taxa indeed have unique transcription patterns, that reflect the temporal (Figs 2 and 3) and spatial (Figs 4 and 5) variability in the environment. The results also support the hypothesis that genetic diversity among microbial strains/ecotypes is reflected in gene expression patterns [19,20]. The MAGC method provides a powerful complementary approach to the WGPB approach to resolve individual gene transcription at the higher taxonomic level, and thereby reveal differential responses that would otherwise be undetectable. The dynamics at the species and sub-species levels underpins a vast array of microbial population genetic diversity. To better understand the fundamental factors that shape marine microbial community composition relative to environmental variability, more highly resolved taxon-specific information is extremely important. Higher resolution approaches may also provide deeper insights into microbiome variability and dynamics in a variety of different environmental contexts.

## Methods

Metatranscriptome sequence data, analyzed in this study, was obtained from NCBI Short Read Archive database (http://www.ncbi.nlm.nih.gov/Traces/sra/) from projects under accession numbers SRA: SRP017469 (the two-day study in the California Current System, CCS [11] and SRA: SRP041215 (three-day study in the North Pacific Subtropical Gyre, NPSG [12]. The probes from the MicroTOOLs microarray [24] were obtained from NCBI Gene Expression Omnibus database (www.ncbi.nlm.nih.gov/geo/) under accession number GPL16706. The probes were further filtered to target sequences that are at least 330-nt long, and the final query database consisted of total 98,632 probes targeting 16,806 gene-OTUs of 145 unique functional genes, where each gene-OTU was targeted by six probes. The probes were designed by Roche NimbleGen (Madison, WI) and quality-checked as described previously [24]. Each set of six 60-nt long probes is specific to one gene-out; one probe could potentially show cross-specificity to another gene-OTU, but not all six probes. It is important to note that probes can be designed for any genes of interest using several approaches, e.g. using eArray (Agilent, earray.chem.agilent.com/earray/). In order to assign each read to a target probe, the probe sequences were used as queries in BLASTN search against the metatranscriptomic sequence databases on a local server with the following parameters: 20,000 hits per query and E value of at least 0.0001. The BLASTN results were sorted to leave only the hits with alignment length of 60 nucleotides and nucleotide identity at least 95% for all probes, except probes for *Prochlorococcus psbA*, for which at least 98% nucleotide identity was selected. Because paired-end Illumina MiSeq sequencing was done in SRP041215, and both pair reads target the same transcript region, only one Illumina read from a pair was selected for further processing. The abundance of a transcript-OTU was calculated as average hit count for all probes (example in Fig 1). Rarely (three instances total, all for the *Prochlorococcus psaA* gene), a read would hit with equal similarity to several probes from different gene-OTUs, and the read was assigned to all these probes. Reads with incorrect assignments showed high variability between the probes and were manually evaluated. Detected transcripts were determined if at least two probes for this transcript had hits in at least one sample, and the minimum total transcription values were at least 3 or 6 (which is equal to 6–36 reads per transcript) in the CCS and NPSG, respectively. Next, gene transcription within each genus was normalized to total read count for this genus in each sample and multiplied by 10^6^.

Transcriptional patterns were defined as the difference in transcript abundance in samples relative to the mean transcript abundance in all samples, and periodic (following a cosine curve over a 24-hour period) transcriptional patterns were identified using the Fourier score (R package ‘cycle’ [47]). The significance of Fourier score was assessed with the false discovery rate (FDR) using a total of 10,000 background models generated with the autoregressive processes of order (AR1) model. The AR1 model allows generating background models with the same distribution as the original dataset [48]. Due to the nature of environmental data, the chosen FDR threshold was higher than it would be for a dataset obtained from cultures, and the significantly periodic transcripts were defined as those that had FDR < 0.25.

Transcriptional patterns were clustered using hierarchical clustering (Pearson correlation for distance measure and complete agglomeration method for clustering), and the significance of cluster assignment was estimated using Approximately Unbiased (AU) P-values of >0.95 (AU P-values range from 0 to 1 and show how strong the cluster is supported by data), where the AU P-values were calculated from at least 1,000 mutliscale bootstrap resampling (pvclust R package [49]). The identified genes, periodicity and cluster assignment for both NPSG and CCS studies are presented in S1 Table. All reads found with the probes for *Prochlorococcus*, *Synechococcus*, SAR11 and *Ostreococcus* are listed in S2 Table.

All data processing and analysis were done using RStudio and BioConductor [50] with the additional packages ggplot2 [51], seqinr [52] and ShortRead [53].

## Supporting Information

S1 File Supporting Tables. Summary of Results Obtained with the MAGC Approach in Comparison to the WGPB Approach for Metatranscriptomes from the Two Oceanic Regions (**Table A**). Genes with Similar Transcriptional Patterns among *Prochlorococcus* OTUs during the 72-h Study in the NPSG (**Table B**). Genes with Similar Transcriptional Patterns among *Synechococcus* OTUs during the 48-h Study in the CCS (**Table C**). Phosphate Concentrations and Primary Production during the NPSG Cruise (**Table D**).
(DOCX)

S1 Fig OTU-specific *Prochlorococcus* Daily Gene Transcription Patterns in the NPSG. *Prochlorococcus* high-light OTUs had similar patterns of transcription for the gene encoding the ammonium transporter (A) *amt* and (B) the *psbA* gene encoding photosystem II core protein. Top panels: Transcriptional composition detected by the MAGC approach, where transcription was normalized to the total *Prochlorococcus* hits in each sample, over time of day (X-axis in hours). OTUs are color-coded according to the heat map coloration below. Middle panels: Hierarchical clustering of transcriptional patterns (by Pearson correlation) for *amt* and *coxA* transcripts. Each row in the heatmap shows transcription of a unique OTU transcript, and each column is a time point within the 72 hour time-series. The OTU transcript ID are only shown, and affiliation for each transcript can be found in S1 Table. Bottom panels: Temporal patterns of total transcript abundances detected by MAGC (open circle) in this study and by WGPB (closed circle) [12] shows that the results of the two approaches are consistent.
(TIF)

S2 Fig OTU-Specific Daily *Synechococcus* Gene Transcription in the CCS. (A) Periodic transcription of the RuBisCO *rbcL* gene and (B) the photosystem I *psbA* genes varied among *Synechococcus* OTUs. Top Panels in each section: Transcriptional composition detected by MAGC, where transcription was normalized to the total *Synechococcus* hits in each sample, over time of day (X-axis in hours). OTUs are color-coded according to the OTU coloration in the heatmap below. Middle panels: Hierarchical clustering of transcriptional patterns (by Pearson correlation) for *rbcL* and *psaA* transcripts. Each row in the heatmap shows transcription of a unique OTU transcript, and each column is a time point within the 48 hour time-series. Bottom panels: Temporal patterns of total transcript abundances detected by MAGC (open circle) in this study and by WGPB (closed circle) [11] shows that the results of the two approaches are consistent.
(TIF)

S3 Fig OTU-Specific SAR11 Daily Gene Transcription in the NPSG. Top Panel: Transcriptional composition detected by MAGC, where transcription was normalized to the total SAR11 hits in each sample, over time of day (X-axis in hours). OTUs are color-coded according to the OTU coloration in the heatmap below. Middle panels: Hierarchical clustering of transcriptional patterns (by Pearson correlation) for *bop* transcripts. Each row in the heatmap shows transcription of a unique OTU transcript, and each column is a time point within the 72 hour time-series. Bottom panels: Temporal patterns of total transcript abundances detected by MAGC (open circle) in this study and by WGPB (closed circle) [12] shows that the results of the two approaches are consistent.
(TIF)

S1 Table Gene Transcription by OTU in the Diel Metatranscriptomes from the NPSG and CCS. Transcription is shown for *Prochlorococcus*, *Synechococcus* and SAR11 genes and for the eukaryotic *rbcL* gene.
(XLSX)

S2 Table Sequence Read IDs Identified by the MAGC Approach from the NPSG and CCS Metatranscriptomes. (XLSX)

## References

[1] Zehr JP, Kudela RM. Nitrogen cycle of the open ocean: from genes to ecosystems. Ann Rev Mar Sci 2011; 3: 197–225. 21329204 10.1146/annurev-marine-120709-142819

[2] Karl DM, Church MJ, Dore JE, Letelier RM, Mahaffey C. Predictable and efficient carbon sequestration in the North Pacific Ocean supported by symbiotic nitrogen fixation. Proc Natl Acad Sci U S A 2012; 109(6): 1842–9. 10.1073/pnas.1120312109 22308450 PMC3277559

[3] Ahlgren NA, Rocap G. Diversity and distribution of marine *Synechococcus*: multiple gene phylogenies for consensus classification and development of qPCR assays for sensitive measurement of clades in the ocean. Front Microbiol. 2012;3: 213 10.3389/fmicb.2012.00213 22723796 PMC3377940

[4] Vergin KL, Beszteri B, Monier A, Thrash JC, Temperton B, Treusch AH, et al High-resolution SAR11 ecotype dynamics at the Bermuda Atlantic Time-series Study site by phylogenetic placement of pyrosequences. ISME J. 2013;7(7): 1322–32. 10.1038/ismej.2013.32 23466704 PMC3695298

[5] Sunagawa S, Coelho LP, Chaffron S, Kultima JR, Labadie K, Salazar G, et al Structure and function of the global ocean microbiome. Science. 2015;348(6237): 1261359 10.1126/science.1261359 25999513

[6] de Vargas C, Audic S, Henry N, Decelle J, Mahé F, Logares R, et al Eukaryotic plankton diversity in the sunlit ocean. Science. 2015;348(6237): 1261605 10.1126/science.1261605 25999516

[7] DeLong EF, Preston CM, Mincer T, Rich V, Hallam SJ, Frigaard N-U, et al Community genomics among stratified microbial assemblages in the ocean's interior. Science. 2006;311(5760): 496–503. 16439655 10.1126/science.1120250

[8] Rusch DB, Halpern AL, Sutton G, Heidelberg KB, Williamson S, Yooseph S, et al The Sorcerer II global ocean sampling expedition: northwest Atlantic through eastern tropical Pacific. PLoS Biol. 2007;5(3): e77 17355176 10.1371/journal.pbio.0050077 PMC1821060

[9] Satinsky BM, Crump BC, Smith CB, Sharma S, Zielinski BL, Doherty M, et al Microspatial gene expression patterns in the Amazon River Plume. Proc Natl Acad Sci U S A. 2014;111(30): 11085–90. 10.1073/pnas.1402782111 25024226 PMC4121788

[10] Kashtan N, Roggensack SE, Rodrigue S, Thompson JW, Biller SJ, Coe A, et al Single-cell genomics reveals hundreds of coexisting subpopulations in wild *Prochlorococcus*. Science. 2014;344(6182): 416–20. 10.1126/science.1248575 24763590

[11] Ottesen EA, Young CR, Eppley JM, Ryan JP, Chavez FP, Scholin CA, et al Pattern and synchrony of gene expression among sympatric marine microbial populations. Proc Natl Acad Sci U S A 2013;110(6): E488–E97. 10.1073/pnas.1222099110 23345438 PMC3568374

[12] Ottesen EA, Young CR, Gifford SM, Eppley JM, Marin R, Schuster SC, et al Multispecies diel transcriptional oscillations in open ocean heterotrophic bacterial assemblages. Science. 2014;345(6193): 207–12. 10.1126/science.1252476 25013074

[13] Aylward FO, Eppley JM, Smith JM, Chavez FP, Scholin CA, DeLong EF. Microbial community transcriptional networks are conserved in three domains at ocean basin scales. Proc Natl Acad Sci U S A. 2015: 201502883. 10.1073/pnas.1502883112 PMC4418921 25775583

[14] Rocap G, Larimer FW, Lamerdin J, Malfatti S, Chain P, Ahlgren NA, et al Genome divergence in two *Prochlorococcus* ecotypes reflects oceanic niche differentiation. Nature. 2003;424(6952): 1042–7. 12917642 10.1038/nature01947

[15] Palenik B, Ren Q, Dupont CL, Myers GS, Heidelberg JF, Badger JH, et al Genome sequence of *Synechococcus* CC9311: insights into adaptation to a coastal environment. Proc Natl Acad Sci U S A. 2006;103(36): 13555–9. 16938853 10.1073/pnas.0602963103 PMC1569201

[16] Martiny AC, Kathuria S, Berube PM. Widespread metabolic potential for nitrite and nitrate assimilation among *Prochlorococcus* ecotypes. Proc Natl Acad Sci U S A. 2009;106(26): 10787–92. 10.1073/pnas.0902532106 19549842 PMC2705535

[17] Malmstrom RR, Rodrigue S, Huang KH, Kelly L, Kern SE, Thompson A, et al Ecology of uncultured *Prochlorococcus* clades revealed through single-cell genomics and biogeographic analysis. ISME J. 2013;7(1): 184–98. 10.1038/ismej.2012.89 22895163 PMC3526172

[18] Konstantinidis KT, DeLong EF. Genomic patterns of recombination, clonal divergence and environment in marine microbial populations. ISME J. 2008;2(10): 1052–1065. 10.1038/ismej.2008.62 18580971

[19] Konstantinidis KT, Serres MH, Romine MF, Rodrigues JLM, Auchtung J, McCue LA, et al Comparative systems biology across an evolutionary gradient within the *Shewanella* genus. Proc Natl Acad Sci U S A. 2009;106(37): 15909–15914. 10.1073/pnas.0902000106 19805231 PMC2747217

[20] Vital M, Chai B, Østman B, Cole J, Konstantinidis KT, Tiedje JM. Gene expression analysis of *E*. *coli* strains provides insights into the role of gene regulation in diversification. ISME J. 2015;9: 1130–1140. 10.1038/ismej.2014.204 25343512 PMC4409157

[21] Philippe N, Crozat E, Lenski RE, Schneider D. Evolution of global regulatory networks during a long-term experiment with *Escherichia coli*. BioAssays 2007;29(9): 846–860. 10.1002/bies.20629 17691099

[22] Eren AM, Maignien L, Sul WJ, Murphy LG, Grim SL, Morrison HG, Sogin ML. Oligotyping: Differentiating between closely related microbial taxa using 16S rRNA gene data. Methods Ecol Evol. 2013;4(12): 1111–1119. 10.1111/2041-210X.12114 PMC3864673 24358444

[23] Eren AM, Morrison HG, Lescault PJ, Reveillaud J, Vineis JH, Sogin ML. Minimum entropy decomposition: Unsupervised oligotyping for sensitive partitioning of highthroughput marker gene sequences. ISME J. 2015;9: 968–979. 10.1038/ismej.2014.195 25325381 PMC4817710

[24] Shilova IN, Robidart JC, Tripp HJ, Turk-Kubo K, Wawrik B, Post AF, et al A microarray for assessing transcription from pelagic marine microbial taxa. ISME J. 2014;8(7): 1476–91. 10.1038/ismej.2014.1 24477198 PMC4069398

[25] Lindell D, Post AF. Ecological aspects of *ntcA* gene expression and its use as an indicator of the nitrogen status of marine *Synechococcus* spp. Appl Environ Microbiol. 2001;67: 3340–3349. 11472902 10.1128/AEM.67.8.3340-3349.2001 PMC93026

[26] Holtzendorff J, Marie D, Post AF, Partensky F, Rivlin A, Hess WR. Synchronized expression of ftsZ in natural *Prochlorococcus* populations of the Red Sea. Environ. Microbiol. 2002;4: 644–653. 12460272 10.1046/j.1462-2920.2002.00347.x

[27] Chen F, Wang K, Kan JJ, Bachoon DS, Lu JR, Lau S et al Phylogenetic diversity of Synechococcus in the Chesapeake Bay revealed by ribulose-1,5-bisphosphate carboxylase-oxygenase (RuBisCO) large subunit gene (rbcL) sequences. Aquat Microb Ecol. 2004;36: 153–164.

[28] Zehr JP, Montoya JP, Jenkins BD, Hewson I, Mondragon E, Short CM et al Experiments linking nitrogenase gene expression to nitrogen fixation in the North Pacific subtropical gyre. Limnol Oceanogr 2007;52: 169–183.

[29] Varaljay VA, Gifford SM, Wilson ST, Sharma S, Karl DM, Moran MA.Bacterial dimethylsulfoniopropionate degradation genes in the oligotrophic north pacific subtropical gyre. Appl Environ Microbiol. 2012;78(8): 2775–82. 10.1128/AEM.07559-11 22327587 PMC3318810

[30] Casciotti K, Ward BB. Dissimilatory nitrite reductase genes from autotrophic ammonia-oxidizing bacteria. Appl Environ Microbiol. 2001;67:2213–2221. 11319103 10.1128/AEM.67.5.2213-2221.2001 PMC92858

[31] Venter JC, Remington K, Heidelberg JF, Halpern AL, Rusch D, Eisen JA, et al Environmental Genome Shotgun Sequencing of the Sargasso Sea. Science 2004;304(5667): 66–74. 15001713 10.1126/science.1093857

[32] Poretsky RS, Hewson I, Sun S, Allen A, Moran MA, Zehr JA. Comparative day/night metatranscriptomic analysis of microbial communities in the North Pacific subtropical gyre. Environ Microbiol 2009;11: 1358–1375. 10.1111/j.1462-2920.2008.01863.x 19207571

[33] Hewson I, Paerl RW, Tripp HJ, Zehr JP, Karl DM. Metagenomic potential of microbial assemblages in the surface waters of the central Pacific Ocean tracks variability in oceanic habitat. Limnol Oceanogr. 2009;55: 1981–1994.

[34] Hewson I, Poretsky RS, Tripp HJ, Montoya JP, Zehr JP. Spatial patterns and light-driven variation of microbial population gene expression in surface waters of the oligotrophic open ocean. Environ Microbiol. 2010;12: 1940–1956. 10.1111/j.1462-2920.2010.02198.x 20406287

[35] Martiny AC, Huang Y, Li W. Occurrence of phosphate acquisition genes in *Prochlorococcus* cells from different ocean regions. Environ Microbiol. 2009;11(6): 1340–7. 10.1111/j.1462-2920.2009.01860.x 19187282

[36] Scanlan DJ, Ostrowski M, Mazard S, Dufresne A, Garczarek L, Hess WR, et al Ecological genomics of marine picocyanobacteria. Microbiol Mol Biol Rev. 2009;73(2): 249–99. 10.1128/MMBR.00035-08 19487728 PMC2698417

[37] Palenik B. Chromatic adaptation in marine *Synechococcus* strains. Appl Environ Microbiol. 2001;67(2): 991–4. 11157276 10.1128/AEM.67.2.991-994.2001 PMC92680

[38] Collier JL, Grossman A. Chlorosis induced by nutrient deprivation in *Synechococcus* sp. strain PCC 7942: not all bleaching is the same. J Bacteriol. 1992;174(14): 4718–26. 1624459 10.1128/jb.174.14.4718-4726.1992 PMC206268

[39] Six C, Thomas J-C, Garczarek L, Ostrowski M, Dufresne A, Blot N, et al Diversity and evolution of phycobilisomes in marine *Synechococcus* spp.: a comparative genomics study. Genome Biol. 2007;8(12): R259 18062815 10.1186/gb-2007-8-12-r259 PMC2246261

[40] Grote J, Thrash JC, Huggett MJ, Landry ZC, Carini P, Giovannoni SJ, et al Streamlining and core genome conservation among highly divergent members of the SAR11 clade. MBio. 2012;3(5): e00252–12. 10.1128/mBio.00252-12 22991429 PMC3448164

[41] Giovannoni SJ, Bibbs L, Cho J-C, Stapels MD, Desiderio R, Vergin KL, et al Proteorhodopsin in the ubiquitous marine bacterium SAR11. Nature. 2005;438(7064): 82–5. 16267553 10.1038/nature04032

[42] Lami R, Kirchman DL. Diurnal expression of SAR11 proteorhodopsin and 16S rRNA genes in coastal North Atlantic waters. Aquat Microb Ecol. 2014;73: 185–94.

[43] Steindler L, Schwalbach MS, Smith DP, Chan F, Giovannoni SJ. Energy starved Candidatus Pelagibacter ubique substitutes light-mediated ATP production for endogenous carbon respiration. PLoS One. 2011;6(5): e19725 10.1371/journal.pone.0019725 21573025 PMC3090418

[44] John DE, López-Díaz JM, Cabrera A, Santiago NA, Corredor JE, Bronk DA, Paul JH. A day in the life in the dynamic marine environment: how nutrients shape diel patterns of phytoplankton photosynthesis and carbon fixation gene expression in the Mississippi and Orinoco River plumes. Hydrobiologia. 2012;679: 155–173.

[45] Robidart JC, Church MJ, Ryan JP, Ascani F, Wilson ST, Bombar D, et al Ecogenomic sensor reveals controls on N2-fixing microorganisms in the North Pacific Ocean. ISME J. 2014;8(6): 1175–85. 10.1038/ismej.2013.244 24477197 PMC4030237

[46] Bombar D, Taylor CD, Wilson ST, Robidart JC, Rabines A, Turk-Kubo KA, et al Measurements of nitrogen fixation in the oligotrophic North Pacific Subtropical Gyre using a free-drifting submersible incubation device. J Plankton Res. 2015;37: 727–739.

[47] Futschik ME. cycle: Significance of periodic expression pattern in time-series data. R package version 1.22.0, 2009. Available: http://itb.biologie.hu-berlin.de/~futschik/software/R/cycle/index.html.

[48] Futschik ME, Herzel H. Are we overestimating the number of cell-cycling genes? The impact of background models on time-series analysis. Bioinformatics. 2008;24: 1063–1069. 10.1093/bioinformatics/btn072 18310054

[49] Suzuki RS, Hidetoshi S. pvclust: Hierarchical Clustering with P-Values via Multiscale Bootstrap Resampling. 2014. Available: http://cran.r-project.org/web/packages/pvclust/index.html.

[50] Gentleman RC, Carey VJ, Bates DM, Bolstad B, Dettling M, Dudoit S, et al Bioconductor: open software development for computational biology and bioinformatics. Genome Biol. 2004;5: R80 15461798 10.1186/gb-2004-5-10-r80 PMC545600

[51] Wickham H. ggplot2: elegant graphics for data analysis. 1st ed New York: Springer; 2009.

[52] Charif D, Lobry JR. SeqinR 1.0–2: A contributed package to the R project for statistical computing devoted to biological sequences retrieval and analysis In: Bastolla U, Porto M, Roman HE, Vendruscolo M, editors. Structural approaches to sequence evolution: Molecules, Networks, Populations. New York: Springer; 2007 pp. 207–232.

[53] Morgan M, Anders S, Lawrence M, Aboyoun P, Pagès H, Gentleman R. ShortRead: a Bioconductor package for input, quality assessment and exploration of high-throughput sequence data. Bioinformatics. 2009;25: 2607–2608. 10.1093/bioinformatics/btp450 19654119 PMC2752612

