# Supplementary material for: Genetic Diversity Affects the Daily Transcriptional Oscillations of Marine Microbial Populations
Source: PLoS One. 2016 Jan 11;11(1):e0146706. doi: 10.1371/journal.pone.0146706 (PMC4709009; doi:10.1371/journal.pone.0146706)
Supplement: S1 File — Summary of Results Obtained with the MAGC Approach in Comparison to the WGPB Approach for Metatranscriptomes from the Two Oceanic Regions (Table A). Genes with Similar Transcriptional Patterns among Prochlorococcus OTUs during the 72-h Study in the NPSG (Table B). Genes with Similar Transcriptional Patterns among Synechococcus OTUs during the 48-h Study in the CCS (Table C). Phosphate Concentrations and Primary Production during the NPSG Cruise (Table D). (DOCX) [file pone.0146706.s001.docx]

**Supporting Information**

**Supporting Tables**

**Table A.** Summary of Results Obtained with the MAGC Approach in Comparison to the WGPB Approach for Metatranscriptomes from the Two Oceanic Regions.

| Organism | Category | WGPB [1,2] | | MAGC (This study) | | |
| --- | --- | --- | --- | --- | --- | --- |
|  |  | Average per sample ± sd | Total | Average per sample ± sd | Total | Reads total as percent of reads identified by WGPB |
| **North Pacific Subtropical Gyre** | | | | | | |
| All | Reads | 4.1±1.0 E+06 | 0.12 E+09^a^ | 22.6±9.5 E+03 | 0.68 E+06 | 0.55 |
| *Prochlorococcus* | Reads | 96±37 E+03 | 2.9 E+06 | 10.6±4.7 E+03 | 0.32 E+06 | 11.0 |
|  | Transcript-OTUs |  | 2.8 E+03 |  | 791^b^ |  |
|  | Genes |  | 2.8 E+03 |  | 54^c^ |  |
| SAR11 | Reads | 26±8 E+03 | 0.77 E+06 | 104±53 | 3.1 E+03 | 0.40 |
|  | Transcript-OTUs |  | 2.8 E+03 |  | 49^b^ |  |
|  | Genes |  | 2.8 E+03 |  | 4^c^ |  |
| **California Current System** | | | | | | |
| All | Reads | 0.77±0.24 E+06 | 1.0 E+07^q^ | 2.9±1.4 E+03 | 3.8 E+04 | 0.38 |
| *Synechococcus* | Reads | 10.5±4.6 E+03 | 0.14 E+06 | 500±200 | 6.5 E+03 | 4.70 |
|  | Transcript-OTUs |  | 4.0 E+03 |  | 98^b^ |  |
|  | Genes |  | 4.0 E+03 |  | 37^c^ |  |
| SAR11 | Reads | 13.5±3.2 E+03 | 0.18 E+06 | 110±38 | 1.4 E+03 | 0.82 |
|  | Transcript-OTUs |  | 1.8 E+03 |  | 83^b^ |  |
|  | Genes |  | 1.8 E+03 |  | 3^c^ |  |
| *Ostreococcus* | *rbcL* reads | 0.97±0.65 E+03 | 12.6 E+03 | 170±88 | 2.2 E+03 | 17.6 |

Nucleotide sequences (reads) detected in this study in all samples, average per sample and as percent of reads detected by WGPB are shown.

^a^ The total read number includes reads for ribosomal RNA

^b^ Number of transcripts detected as described in the Methods.

^c^ Number of genes with detected transcription (for example, *psaA, psbA, coxA*).

**Table B.** Genes with Similar Transcriptional Patterns among *Prochlorococcus* OTUs during the 72-h Study in the NPSG.

| **Gene** | **Annotation** | **KEGG Pathway** | **OTU-transcripts detected** |
| --- | --- | --- | --- |
| *amt* | ammonium transporter | Membrane transport | 16 |
| *coxA* | cytochrome c oxidase subunit I | Energy metabolism (oxidative phosphorylation) | 15 |
| *coxB* | cytochrome c oxidase subunit II | Energy metabolism (oxidative phosphorylation) | 7 |
| *fur* | Fur family transcriptional regulator; Ferric uptake regulator | Transcription factors | 9 |
| *glnA* | glutamine synthetase, type I | Amino acid metabolism | 2 |
| *kaiC* | circadian clock protein KaiC | [Circadian clock] | 8 |
| *sodN* | nickel-containing superoxide dismutase (NiSOD) | [Oxidative stress response] | 2 |
| *phrB* | similar to DNA photolyase | Replication and repair | 5 |
| *pip* | proline iminopeptidase | Amino acid metabolism | 10 |
| *pmm1148* | possible EF-1 guanine nucleotide exchange domain | Nucleotide metabolism | 9 |
| *pmm1359* | predicted membrane protein PMM1359 | Membrane transport | 9 |
| *psaB* | photosystem I P700 chlorophyll a apoprotein A10 | Energy metabolism (photosynthesis) | 28 |
| *psbA* | photosystem II PsbA protein (D1) | Energy metabolism (photosynthesis) | 15 |
| *ptox* | plastoquinol terminal oxidase | Energy metabolism | 5 |
| *rbcL* | RuBisCO, large chain | Carbon metabolism (carbon fixation) | 3 |
| *urtA* | urea substrate binding transporter protein | Membrane transport | 13 |
| **SUM** | **16** |  | **161** |

For each gene, number of reads identified using MAGC for all samples and as a percent relative to the number of reads identified by WGPB [2], correlation (the Pearson correlation coefficient) between results obtained by the two approaches and number of transcript-OTUs identified in all samples are shown. In the KEGG pathway column, the square brackets indicate pathways/metabolisms/processes that do not have KEGG assignment.

**Table C.** Genes with Similar Transcriptional Patterns among *Synechococcus* OTUs during the 48-h Study in the CCS.

| **Gene** | **Annotation** | **KEGG Pathway** | **OTU-transcripts detected** |
| --- | --- | --- | --- |
| *amt* | ammonium transporter | Membrane transport | 3 |
| *cpaB2* | phycocyanin, beta subunit | Energy metabolism (photosynthesis) | 2 |
| *dxs* | 1-deoxy-D-xylulose-5-phosphate synthase | Carbohydrate and lipid metabolism | 7 |
| *glnA* | glutamine synthetase, type I | Amino acid metabolism | 4 |
| *idiA* | iron ABC transporter, substrate binding protein | Membrane transport | 6 |
| *isiB* | flavodoxin | Energy metabolism (photosynthesis) | 3 |
| *kaiC* | circadian clock protein KaiC | [Circadian clock] | 7 |
| *phoH* | phosphate starvation-inducible protein | [Phosphorus stress response] | 3 |
| *pmm1148* | possible EF-1 guanine nucleotide exchange domain | Nucleotide metabolism | 2 |
| *pyk* | pyruvate kinase | Carbon metabolism (glycolysis) | 5 |
| *sodC* | copper/zinc superoxide dismutase | [Oxidative stress response] | 3 |
| **SUM** | **11** |  | **55** |

For each gene, number of reads identified using MAGC for all samples and as a percent relative to the number of reads identified by WGPB [1], correlation (the Pearson correlation coefficient) between results obtained by the two approaches and number of transcript-OTUs identified in all samples are shown. In the KEGG pathway column, the square brackets indicate pathways/metabolisms/processes that do not have KEGG assignment.

**Table D. Phosphate Concentrations and Primary Production during the NPSG Cruise.** Concentrations of phosphate were obtained from Robidart *et al.* [3], and primary production rates were obtained from Bombar *et al.* [4]. Primary production had a significant positive relationship with phosphate concentrations over the course of the NPSG cruise (r=0.38, n=5).

| Date | Phosphate (μmol) | Primary production (nmol C L^-1^ day^-1^) |
| --- | --- | --- |
| 9/10/11 | 0.017 | 229 |
| 9/11/11 | 0.127 | 237 |
| 9/13/11 | 0.099 | 135 |
| 9/16/11 | 0.143 | 330 |
| 9/18/11 | 0.118 | 400 |

**References**

1. Ottesen EA, Young CR, Eppley JM, Ryan JP, Chavez FP, Scholin CA, et al. Pattern and synchrony of gene expression among sympatric marine microbial populations. Proc Natl Acad Sci U S A. 2013;110(6): E488-E97.
2. Ottesen EA, Young CR, Gifford SM, Eppley JM, Marin R, Schuster SC, et al. Multispecies diel transcriptional oscillations in open ocean heterotrophic bacterial assemblages. Science. 2014;345(6193): 207-12.
3. Robidart JC, Church MJ, Ryan JP, Ascani F, Wilson ST, Bombar D, et al. Ecogenomic sensor reveals controls on N2-fixing microorganisms in the North Pacific Ocean. ISME J. 2014;8(6): 1175-85.
4. Bombar D, Taylor CD, Wilson ST, Robidart JC, Rabines A, Turk-Kubo KA, et al. Measurements of nitrogen fixation in the oligotrophic North Pacific Subtropical Gyre using a free-drifting submersible incubation device. J Plankton Res. 2015;37: 727-739.
