# Supplementary figures and images for: Genetic Diversity Affects the Daily Transcriptional Oscillations of Marine Microbial Populations
Source: PLoS One. 2016 Jan 11;11(1):e0146706. doi: 10.1371/journal.pone.0146706 (PMC4709009; doi:10.1371/journal.pone.0146706)

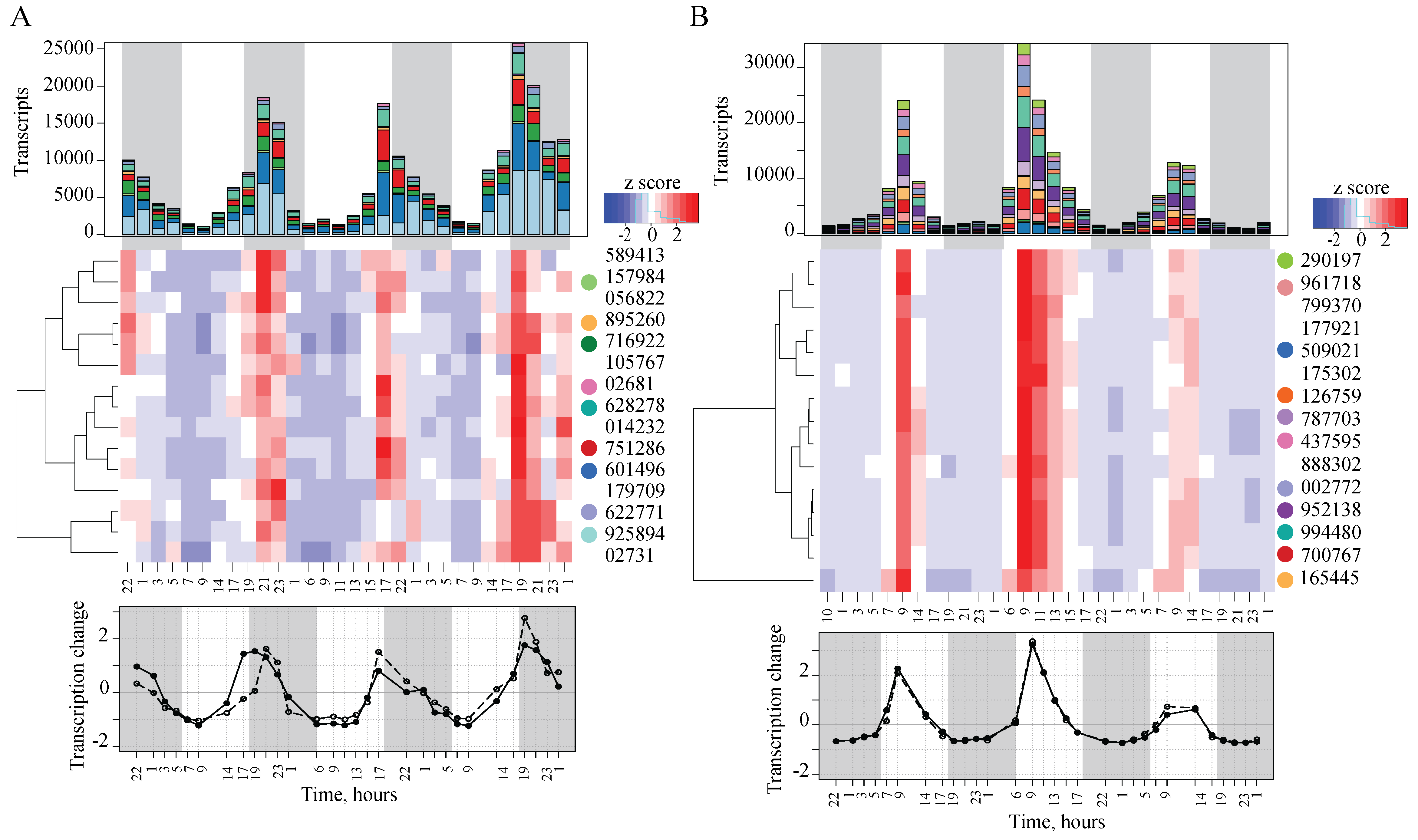

Supplement: S1 Fig — Prochlorococcus high-light OTUs had similar patterns of transcription for the gene encoding the ammonium transporter (A) amt and (B) the psbA gene encoding photosystem II core protein. Top panels: Transcriptional composition detected by the MAGC approach, where transcription was normalized to the total Prochlorococcus hits in each sample, over time of day (X-axis in hours). OTUs are color-coded according to the heat map coloration below. Middle panels: Hierarchical clustering of transcriptional patterns (by Pearson correlation) for amt and coxA transcripts. Each row in the heatmap shows transcription of a unique OTU transcript, and each column is a time point within the 72 hour time-series. The OTU transcript ID are only shown, and affiliation for each transcript can be found in S1 Table. Bottom panels: Temporal patterns of total transcript abundances detected by MAGC (open circle) in this study and by WGPB (closed circle) [12] shows that the results of the two approaches are consistent. (TIF) [file pone.0146706.s002.tif]

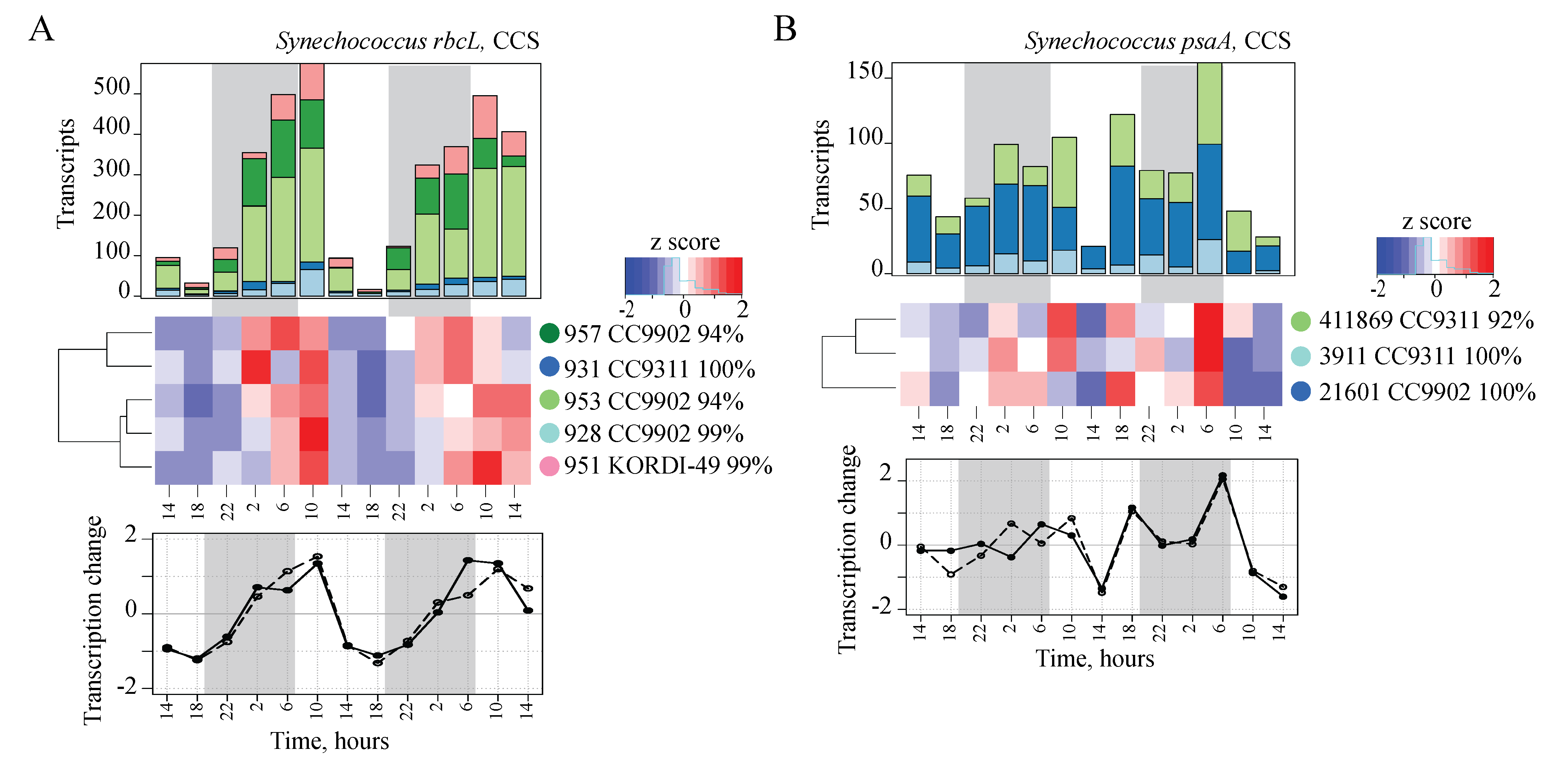

Supplement: S2 Fig — (A) Periodic transcription of the RuBisCO rbcL gene and (B) the photosystem I psbA genes varied among Synechococcus OTUs. Top Panels in each section: Transcriptional composition detected by MAGC, where transcription was normalized to the total Synechococcus hits in each sample, over time of day (X-axis in hours). OTUs are color-coded according to the OTU coloration in the heatmap below. Middle panels: Hierarchical clustering of transcriptional patterns (by Pearson correlation) for rbcL and psaA transcripts. Each row in the heatmap shows transcription of a unique OTU transcript, and each column is a time point within the 48 hour time-series. Bottom panels: Temporal patterns of total transcript abundances detected by MAGC (open circle) in this study and by WGPB (closed circle) [11] shows that the results of the two approaches are consistent. (TIF) [file pone.0146706.s003.tif]

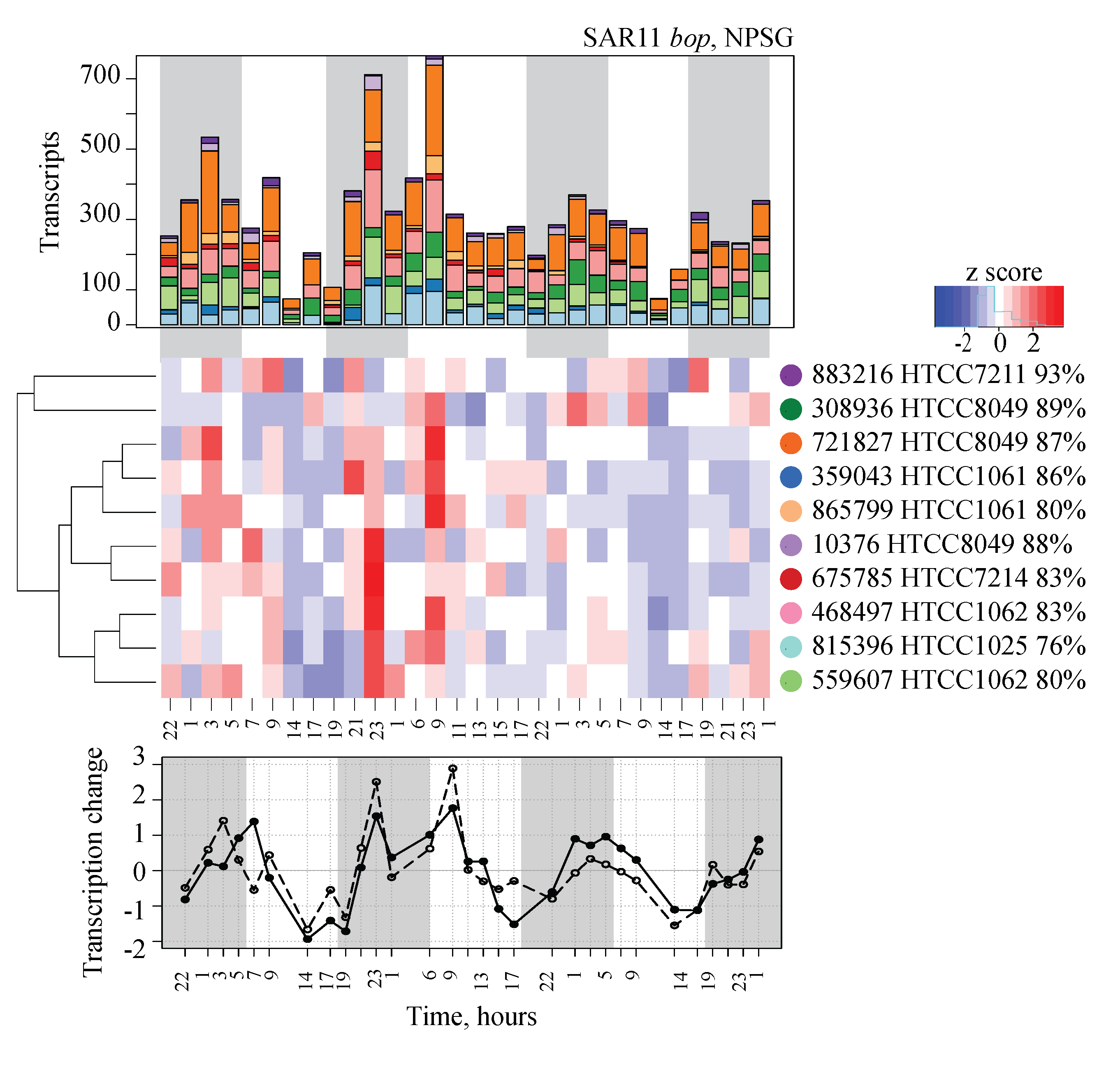

Supplement: S3 Fig — Top Panel: Transcriptional composition detected by MAGC, where transcription was normalized to the total SAR11 hits in each sample, over time of day (X-axis in hours). OTUs are color-coded according to the OTU coloration in the heatmap below. Middle panels: Hierarchical clustering of transcriptional patterns (by Pearson correlation) for bop transcripts. Each row in the heatmap shows transcription of a unique OTU transcript, and each column is a time point within the 72 hour time-series. Bottom panels: Temporal patterns of total transcript abundances detected by MAGC (open circle) in this study and by WGPB (closed circle) [12] shows that the results of the two approaches are consistent. (TIF) [file pone.0146706.s004.tif]
